# Seroepidemiological Analysis of Canine Leptospira Species Infections in Changchun, China

**DOI:** 10.3390/pathogens12070930

**Published:** 2023-07-12

**Authors:** Yue Ding, Wenlong Zhang, Xufeng Xie, Shilei Zhang, Ning Song, Zhanbin Liu, Yongguo Cao

**Affiliations:** 1 State Key Laboratory for Diagnosis and Treatment of Severe Zoonotic Infectious Diseases, Key Laboratory for Zoonosis Research of the Ministry of Education, Institute of Zoonosis, College of Veterinary Medicine, Jilin University, Changchun 130062, China; 2 Department of Clinical Veterinary Medicine, College of Veterinary Medicine, Jilin University, Changchun 130062, China; 3 Nanchang Police Dog Base of the Ministry of Public Security, Nanchang 330100, China

**Keywords:** dog, canine leptospirosis, seroepidemiology, Changchun, China

## Abstract

Leptospirosis is a significant worldwide zoonotic infectious disease that infects a wide range of animals and humans. *Leptospira* will colonize the animal’s urinary and reproductive systems and be excreted with urine, potentially causing a wide range of infections. Dogs are an essential host for *Leptospira*, and epidemiological investigation studies of leptospirosis must be conducted to clarify the prevalence of leptospirosis and to reduce the risk of transmission to humans. This study aimed to investigate the seroepidemiology of leptospiral infection in dogs from Changchun, China, using Microscopic Agglutination Test (MAT). A total of 1053 canine blood samples were collected and tested by MAT. The positive rate of MAT was approximately 19.1%. The main prevalent *Leptospira* serogroups were *L.* Icterohaemorrhagiae (8.1%), *L.* Canicola (7.6%), *L*. Australis (5.3%), *L*. Ballum (4.7%) and *L*. Pyrogenes (4.2%). No statistically significant difference among different varieties, sexes and sampling seasons (*p* > 0.05), except the age (*p* < 0.05). The seropositive rate was much higher in adult and aged dogs than in juvenile dogs. Our results showed the seroprevalence and the prevalent serogroup of Canine leptospirosis in Changchun, China.

## 1. Introduction

Leptospirosis is a widely spread zoonotic disease caused by pathogenic leptospiral infection, and more than 200 animals, as well as humans, can be infected [1]. Mild conditions often present with fever and, like influenza, severe infections can present with multisystem impairments and, in extreme cases, are life-threatening [2,3]. Leptospira will reproduce continuously within the urinary and reproductive systems of animals, following the discharge of urine into the environment, where it can survive for months in a humid climate and is highly infectious [4,5]. Leptospirosis is transmitted to humans by direct or indirect contact with the urine of infected animals [6,7]. Leptospirosis is endemic in tropical and subtropical regions of Asia, Oceania and South America [7,8]. Still, it has also been frequently reported in recent years in Europe, North America and Africa, among others [9,10,11]. At present, the prevalence of leptospirosis is expanding, and the endemic locations are not limited to tropical regions, so epidemiological investigation of leptospirosis and precise regional distribution of *Leptospira* serogroup are of great importance for the prevention of leptospirosis.

Dogs are the primary hosts for Canicola and Icterohaemorrhagiae serovars [12,13]. Infection in dogs may cause variable symptoms; moreover, some dogs may have mild or no signs of infection, and sometimes they may die [7,14]. Asymptomatic and chronic carriers may also become infectious agents [15]. People are in close contact with dogs, and infected dogs have the potential risk of transmitting *Leptospira* to humans [16,17]. So, Canine leptospirosis monitoring is beneficial to human leptospirosis prevention and control.

Laboratory diagnosis of leptospirosis can be made by several methods, including direct microscopic examination and culture, as well as serological and molecular methods, such as Polymerase Chain Reaction (PCR) [18]. The Microscopic Agglutination Test (MAT) is the gold standard serological test for leptospirosis and estimates the presence or absence of antibodies in the canine serum [2,19]. The MAT can recognize infected bacterial sera, facilitating the discovery of a possible source of infection in animals [20,21]. The MAT tests target leptospiral antibodies in serum, not *Leptospira* strains.

There are only a few epidemiological reports of canine leptospirosis in China, most based on surveys of cities in the south [22,23], which is consistent with the fact that leptospirosis occurs mainly in warm and humid parts of the south [24,25]. Changchun is located in the subtropical region of China. Although the number of canines is large, the epidemiological investigation of canine leptospirosis is lacking. In this study, the seroepidemiological analysis of Canine leptospirosis was conducted by MAT in Changchun, China.

## 2. Material and Methods

### 2.1. Study Area and Study Population

**Figure 1:**
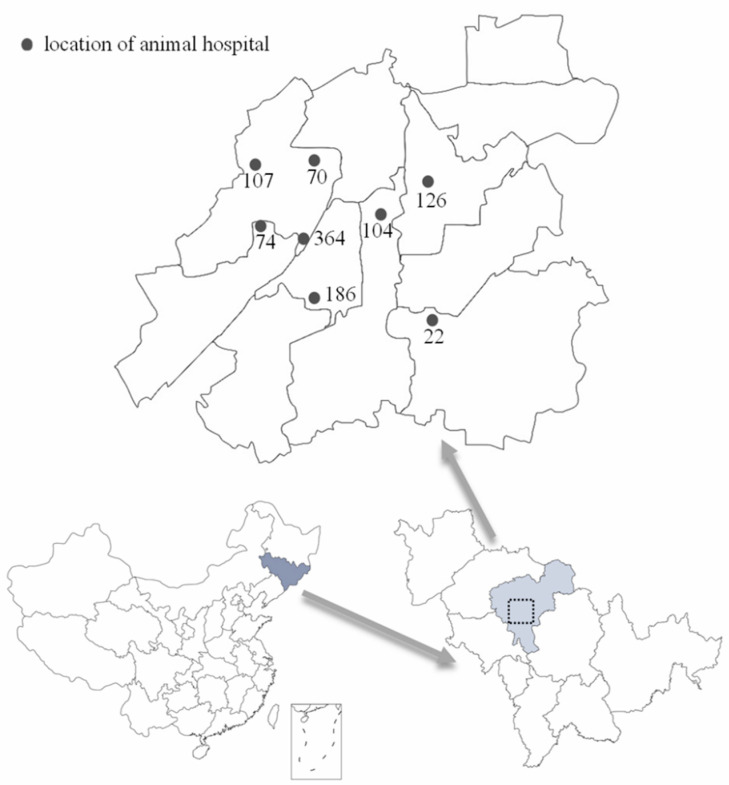
The geographical location of Changchun City, Jilin Province and the location distribution of animal hospitals for sample collecting (gray dots). From June 2020 to May 2021, 1053 canine blood samples were collected from eight animal hospitals.

The study was conducted in Changchun, China. Changchun is the capital city of Jilin Province; the city has a latitude and longitude of 43°88′ N, 125°32′ E, and an elevation of 250–350 m. Changchun has a temperate monsoon climate with high temperatures and rain in summer. We collaborated with eight animal hospitals from June 2020 to May 2021 to collect canine blood (Figure 1). Information provided with canine blood included breed, sex, age, the time of blood collection, and the vaccine injection information for some dogs. No information could be determined about the dog owner. Therefore confidentiality was protected. Samples were excluded from the study with incomplete records for quality control checks or duplicate samples from a single dog.

### 2.2. Sample Preparation

The dog’s blood was obtained by sterile venipuncture, collected in a vacuum blood collection tube (anticoagulant EDTA), and placed in a 4 °C freezer. Then, the sample was transferred to the laboratory. Samples were centrifuged at 3000 r for 10 min; serum was separated and stored at −20 °C.

### 2.3. Microscopic Agglutination Test (MAT) 

Fifteen *Leptospira* representing fifteen different serogroups were included in this study (see Supplementary Table S1). All *Leptospira* were used for the microscopic agglutination test (MAT). Leptospira was grown in liquid Ellinghausen–McCullough–Johnson–Harris (EMJH) medium at 29 °C. The sera were diluted to 1:100 with phosphate-buffered saline (PBS). The serum samples were added to the live *Leptospira* cell suspensions in 96-well round-bottomed microtiter plates at room temperature in the dark for 2 h [26]. Negative and positive sera by adding live antigens were used as the control. Subsequently, an aliquot from wells was added on a slide and observed under 20× magnification using dark field microscopy (Olympus CX43RF, Tokyo, Japan). Every serum that gives an agglutination of at least 50% of the *Leptospira* or agglutination may occur, compared to the negative control, is considered positive [27]. 

### 2.4. Statistical Analyses

Data were organized, summarized, and then analyzed using GraphPad Prism 9. Results with *p* values < 0.05 were considered statistically significant. The Chi-square test (χ^2^) was used to determine whether there is a substantial association in occurrences of leptospiral species antibodies in dogs according to varieties, age, sex, and season.

### 2.5. Ethical Issue

The purpose of the study was clarified to the dog’s owners and the family members, and then verbal informed consent was obtained from dog owners before blood sample collection. The design and process of the experiment strictly abide by the relevant national ethical principles of experimental animals, with the permission of the company’s Experimental Animal Management Committee and Ethics Committee, and carried out following the regulations of the Administration of Affairs Concerning Experimental Animals in China. The protocol was approved by the Institutional Animal Care and Use Committee of Jilin University (20170318).

## 3. Result

### 3.1. Sample Collection Results and Overall Seroprevalence

A total of 1053 canine blood samples were collected from June 2020 to June 2021 in Changchun pet hospital. There were 527 male and 526 female dogs in the sample population. Two hundred three dogs under one year old, 393 dogs 1–7 years old, and 457 dogs over seven years old (Table 1). The breeds of dogs were Poodle, Golden Retriever, Chinese rural dog, etc. Sampling times were counted by season (Table 2). There were 201 serums of 1053 tested positive by MAT, reflecting a seroprevalence of 19.1%. The main positive serogroups were *L*. Icterohaemorrhagiae (8.1%), *L*. Canicola (7.6%), *L*. Ballum (4.7%), *L*. Pyrogenes (4.2%) and *L*. Australis (5.3%), as presented in Table 3. Among the positive samples, there are 98 of 201 (48.76%) tested positive with at least two serogroups.

### 3.2. Association between Seropositive Rate and Risk Factors

There was a significant association between canine age and the seropositive rate (*p* < 0.05). Dogs less than one-year-old had the lowest positive rate. Dogs over the age of 1 year are more susceptible to leptospiral infection. The number of positive samples was the lowest in younger dogs (within one year). There was no significant association between seroprevalence and breed, sex, or sampling time (Table 4). Although female dogs had more positive samples than male dogs, it was not statistically significant (*p* > 0.05). 

### 3.3. Effect of Leptospirosis Vaccine on Positive Samples

Of the total sample, 105 had been vaccinated against *Leptospira* within a year. Leptospira vaccine designations were replaced by LV-I, LV-II, LV-III and LV-IV; the number of samples was 36, 21, 45, and 3, respectively (Table 5). All of the vaccines contained the leptospiral serotypes Canicola and Icterohaemorrhagiae. Anti-leptospira antibodies were detected in 58 samples of 105. The vaccine immunization success rate is approximately 55.2%. The immunization success rate is higher in LV-I and LV-IV groups than in LV-II and LV-III groups (Table 5).

### 3.4. Excluding the Vaccine Factor, the Favorable Serogroups Distribution

**Figure 2:**
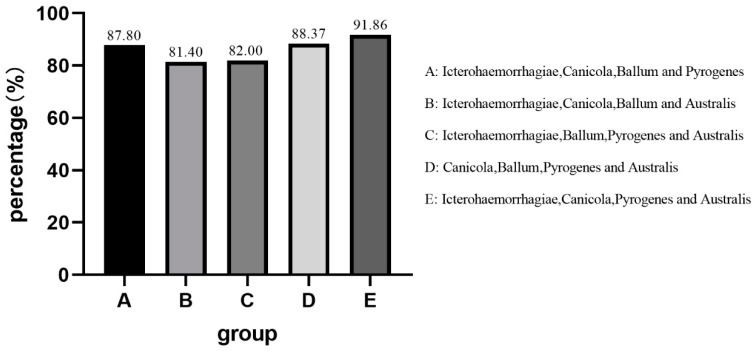
Different serogroup combinations. The main positive serogroups, Icterohaemorrhagiae, Canicola, Ballum, Pyrogenes and Australis, were combined in permutations, with four serogroups as one group. The sum of the positive rate in each group was calculated.

Among the 58 immune samples, there are 29 samples tested positive, with only *L*. Icterohaemorrhagiae and *L*. Canicola serogroups. These 29 samples were discarded, leaving 172 of 1024 positive sera. The main positive serogroups were *L*. Icterohaemorrhagiae, *L*. Canicola, *L*. Ballum, *L*. Pyrogenes and *L*. Australis (Table 6). These five serogroups were combined in permutations, with four serogroups as one group. Corresponding to the combined serogroups group, the number of samples containing one or more serogroups will be counted and compared with the total samples. The results showed that serogroups group E had the highest proportion (Figure 2). If the serogroups of *the Leptospira* vaccine were made up of *L*. Icterohaemorrhagiae, *L*. Canicola, *L*. Pyrogenes and *L*. Australis, the protection rate might reach 91.86% theoretically (Figure 2).

## 4. Discussion

Leptospirosis is considered to be an essential worldwide zoonotic disease [28]. Little studies have been carried out on Canine leptospirosis in Changchun, China. MAT conducted the seroepidemiological analysis of Canine leptospirosis in this study. According to MAT test results, 201 of 1053 dogs tested positive. Dogs also MAT test positive when infected with non-pathogenic *Leptospira* or subclinical infection. In this experiment, only antibodies in dog serum were detected. The MAT seroprevalence of dogs with *Leptospira* in Changchun was 19.1%. Our results are slightly lower than those of other countries [29,30]. In a study conducted in the city of Mashhad in 2003, the *Leptospira* seroprevalence in dogs was estimated at 41.6% [31], much higher than the rate in our study (19.1%). The possible reason is that Changchun is temperate, and others are tropical or subtropical. *Leptospira* spp. are more likely to survive in warmer environments.

There were 103 positive cases in female dogs and more than 98 positive cases in male dogs, but there was no statistical difference (Table 4). Studies have shown that the risk of leptospirosis in female dogs is significantly higher than in male dogs in other areas. Surveys have also shown significantly higher seropositive rates in male dogs because male dogs are thought to be more likely to roam and, thus, be exposed to infected environments [15]. There was a statistical difference between the age of dogs and the positive rate (*p* < 0.05). Seropositivity in dogs 1–7 years and over seven years is much higher than in dogs less than one-year-old (Table 4). This may be due to juvenile animals being bundle bound and less likely to roam or engage in activities that could lead to natural infections, thus reducing exposure. In New Zealand, the positive rate of leptospirosis is significantly lower in dogs over 12 years of age, which is associated with decreased physical function, vitality, and reduced exposure to infectious agents in older dogs [15]. In general, the greater the dog’s range, the higher the likelihood of exposure to the source of infection, the more susceptible to infection. Surveys in several countries of the European region have shown that the season of high incidence is summer and autumn [32,33]. In this study, the positive rate was higher in spring and autumn, but there was no significant difference.

It is generally accepted that *L*. Icterohaemorrhagiae and *L*. Canicola are the predominant serogroups of canine infection [12]. In our study, the other three serogroups, *L*. Australis, *L*. Ballum and *L*. Pyrogenes, accounted for more than half of the positive samples and should be concerned. Although pathogenic *L.* Pomona and *L.* Pyrogenes have not been reported in China, our team successfully isolated the *L.* Australis strain of Leptospirosis from an infected dog in 2020 [24]. These three types of *Leptospira* are likely to become potential pathogens and threaten dogs’ health. Humans are closely related to dogs, and dogs, as the storage host of *Leptospira*, may transmit it to humans and affect human health. Therefore, it is necessary to pay attention to the prevalence of *Leptospira*. Recently, new vaccines containing an expanded spectrum of three or four serogroups (Icterohaemorrhagiae, Canicola, Grippotyphosa and/or Bratislava) have been introduced to the market [34]. Our data indicated that the vaccine serogroups used were not matched with the epidemic serogroups in Changchun. This study sheds light on *Leptospira* vaccine serogroup selection using *L.* Icterohaemorrhagiae, *L.* Canicola, *L.* Pyrogenes and *L.* Australis.

## Figures and Tables

**Table 1:** Dog age and sex statistics.

Age	Number		Total
	Male	Female	
<1 years	106	97	203
1–7 years	201	192	393
>7 years	220	237	457
Total	527	526	1053

**Table 2:** Dog breeds, seasonal distribution statistics.

Breed	Number				Total
	Spring	Summer	Autumn	Winter	
Poodle	45	102	57	54	258
Chinese rural dog	22	61	37	23	143
Golden Retriever	14	38	27	10	89
Bichon Frise	6	43	26	10	85
Pomeranian	10	30	10	11	61
Labrador Retriever	7	24	9	5	45
Bulldog	7	19	10	6	42
Samoyed	9	12	13	6	40
All other breeds	38	128	77	47	290
Total	158	457	266	172	1053

**Table 3:** Statistics of positive samples of each serogroup.

Serogroups	No. Positive	Positivity Rate (% of Positive Samples) ^a^	Positivity Rate (% of All Samples) ^b^
Icterohaemorrhagiae	85	42.3	8.1
Javanica	12	6.0	1.1
Canicola	80	39.8	7.6
Ballum	49	24.4	4.7
Pyrogenes	44	21.9	4.2
Autumnalis	6	3.0	0.6
Australis	56	27.9	5.3
Hebdomadis	11	5.5	1.0
Paidjan	2	1.0	0.2

^a^ The number of positive samples for each serogroup represented the percentage of the total positive samples (201). ^b^ The number of positive samples for each serogroup represented the percentage of the total samples (1053).

**Table 4:** Relationship between possible risk factors and sample positivity.

Variable	Level	Number (*n* = 1053)	No. Positive	Positivity Rate (%)	χ^2^	*p*-Value
Sex					0.166	0.684
	Female	526	103	19.6		
	Male	527	98	18.6		
Age					33.33	<0.0001
	<1 year	203	10	4.9		
	1–7 year	393	93	23.7		
	>7 year	457	98	21.4		
Breed					8.811	0.358
	Poodle	258	60	23.3		
	Chinese rural dog	143	22	15.4		
	Golden Retriever	89	15	16.9		
	Bichon Frise	85	19	22.4		
	Pomeranian	61	9	14.8		
	Labrador Retriever	45	7	15.6		
	Bulldog	42	11	26.2		
	Samoyed	40	9	22.5		
	All other breeds	290	49	16.7		
Season					3.841	0.279
	Spring	158	35	22.2		
	Summer	457	77	16.9		
	Autumn	266	58	21.8		
	Winter	172	31	18.0		

**Table 5:** The number of samples and rate of production of corresponding leptospiral antibodies after commercial vaccination of dogs against *Leptospira* spp.

Vaccine	Number	No. Positive (% of Level)
LV-I	36	23 (63.9)
LV-II	21	11 (52.4)
LV-III	45	22 (49.0)
LV-IV	3	2 (66.7)

**Table 6:** Number and proportion of samples with MAT positive after excluding vaccine effects.

Serogroups	No. Positive	Positivity Rate (%) ^a^	Overall Positive Rate (%) ^b^
Icterohaemorrhagiae	56	32.7	5.5
Javanica	12	7.0	1.2
Canicola	52	30.2	5.1
Ballum	49	28.5	4.8
Pyrogenes	44	25.6	4.3
Autumnalis	6	3.5	0.6
Australis	56	32.6	5.5
Hebdomadis	11	6.4	1.2
Paidjan	2	1.2	0.2

^a^ After excluding the 29 samples that produced only *L*. Icterohaemorrhagiae and *L*. Canicola antibodies, the total number of positive samples was 172. The positive samples of each serogroup accounted for the percentage of the total positive samples. ^b^ After excluding the 29 samples that produced only *L*. Icterohaemorrhagiae and *L*. Canicola antibodies, the total number of samples was 11,024. The positive samples of each serogroup accounted for the percentage of total samples.

## Data Availability

All data are presented in this article.

## Supplementary Materials

The following supporting information can be downloaded at: https://www.mdpi.com/article/10.3390/pathogens12070930/s1, Table S1: *Leptospira* strain used in MAT.

## References

[1] Adler B. (2015). Leptospira and Leptospirosis, in Current Topics in Microbiology and Immunology.

[2] Rajapakse S. (2022). Leptospirosis: Clinical aspects. Clin. Med..

[3] Picardeau M. (2020). Leptospira and Leptospirosis. Methods Mol. Biol..

[4] McCallum K.E., Constantino-Casas F., Cullen J.M., Warland J.H., Swales H., Linghley N., Kortum A.J., Sterritt A.J., Cogan T., Watson P.J. (2019). Hepatic leptospiral infections in dogs without obvious renal involvement. J. Vet. Intern. Med..

[5] Alton G.D., Berke O., Reid-Smith R., Ojkic D., Prescott J.F. (2009). Increase in seroprevalence of canine leptospirosis and its risk factors, Ontario 1998–2006. Can. J. Vet. Res..

[6] Akhvlediani T., Bautista C.T., Garuchava N., Sanodze L., Kokaia N., Malania L., Chitadze N., Sidamonidze K., Rivard R.G., Hepburn M.J. (2017). Epidemiological and Clinical Features of Brucellosis in the Country of Georgia. PLoS ONE.

[7] Marami L.M., Gebremedhin E.Z., Sarba E.J., Tola G.K., Endalew S.S., Tesfaye A.M., Presti V.D.M.L., Vitale M. (2021). Seroprevalence and Associated Risk Factors of Canine Leptospira and Brucella Species Infection in West Shewa Zone, Central Ethiopia. Vet. Med..

[8] Kurilung A., Chanchaithong P., Lugsomya K., Niyomtham W., Wuthiekanun V., Prapasarakul N. (2017). Molecular detection and isolation of pathogenic Leptospira from asymptomatic humans, domestic animals and water sources in Nan province, a rural area of Thailand. Res. Vet. Sci..

[9] Smith J.K.G., Young M.M., Wilson K.L., Craig S.B. (2013). Leptospirosis following a major flood in Central Queensland, Australia. Epidemiol. Infect..

[10] Miraglia F., Matsuo M., Morais Z.M., Dellagostin O.A., Seixas F.K., Freitas J.C., Hartskeerl R., Moreno L., Costa B.L., Souza G.O. (2013). Molecular characterization, serotyping, and antibiotic susceptibility profile of Leptospira interrogans serovar Copenhageni isolates from Brazil. Diagn. Microbiol. Infect. Dis..

[11] Traxler R.M., Callinan L.S., Holman R.C., Steiner C., Guerra M.A. (2014). Leptospirosis-Associated Hospitalizations, United States, 1998–2009. Emerg. Infect. Dis..

[12] Stull J.W., Evason M., Weese J.S., Yu J., Szlosek D., Smith A.M. (2022). Canine leptospirosis in Canada, test-positive proportion and risk factors (2009 to 2018): A cross-sectional study. PLoS ONE.

[13] Browne E.S., Callefe J.L.R., DE Jesus E.R., Zeppelini C.G., Cremonese C., Costa F. (2022). A Systematic Review of the geographic distribution of pathogenic Leptospira serovars in the Americas, 1930–2017. An. Acad. Bras. Ciências.

[14] Miotto B.A., Guilloux A.G.A., Tozzi B.F., Moreno L.Z., Da Hora A.S., Dias R.A., Heinemann M.B., Moreno A.M., Filho A.F.D.S., Lilenbaum W. (2018). Prospective study of canine leptospirosis in shelter and stray dog populations: Identification of chronic carriers and different Leptospira species infecting dogs. PLoS ONE.

[15] Harland A., Cave N., Jones B., Benschop J., Donald J., Midwinter A., Squires R.A., Collins-Emerson J. (2013). A serological survey of leptospiral antibodies in dogs in New Zealand. N. Z. Vet. J..

[16] Nomura A., Imaoka K., Imanishi H., Shimizu H., Nagura F., Maeda K., Tomino T., Fujita Y., Kimura M., Stein G.H. (2010). Human *Brucella canis* Infections Diagnosed by Blood Culture. Emerg. Infect. Dis..

[17] Lucero N.E., Corazza R., Almuzara M.N., Reynes E., Escobar G.I., Boeri E., Ayala S.M. (2010). Human Brucella canis outbreak linked to infection in dogs. Epidemiol. Infect..

[18] Samrot A.V., Sean T.C., Bhavya K.S., Sahithya C.S., Chan-Drasekaran S., Palanisamy R., Robinson E.R., Subbiah S.K., Mok P.L. (2021). Leptospiral Infection, Pathogenesis and Its Diagnosis—A Review. Pathogens.

[19] Goldstein R.E. (2010). Canine Leptospirosis. Vet. Clin. N. Am. Small Anim. Pract..

[20] Harkin K.R., Roshto Y.M., Sullivan J.T., Purvis T.J., Chengappa M.M. (2003). Comparison of polymerase chain reaction assay, bacteriologic culture, and serologic testing in assessment of prevalence of urinary shedding of leptospires in dogs. J. Am. Vet. Med. Assoc..

[21] Lizer J., Grahlmann M., Hapke H., Velineni S., Lin D., Kohn B. (2017). Evaluation of a rapid IgM detection test for diagnosis of acute leptospirosis in dogs. Vet. Rec..

[22] Hu W., Lin X., Yan J. (2014). Leptospira and leptospirosis in China. Curr. Opin. Infect. Dis..

[23] Yalin W., Lingbing Z., Hongliang Y., Jianmin X., Xiangyan Z., Xiaokui G., Utpal P., Jinhong Q. (2011). High prevalence of pathogenic Leptospira in wild and domesticated animals in an endemic area of China. Asian Pac. J. Trop. Med..

[24] Song N., Zhang W., Ding Y., Wu D., Dai Z., Xu L., Cao Y. (2020). Preliminary Characterization of Dog Derived Pathogenic Strains of Leptospira interrogans Serovar Australis in Nanchang of Jiangxi Province, China. Front. Vet. Sci..

[25] Zhang C., Xu J., Zhang T., Qiu H., Li Z., Zhang E., Li S., Chang Y.-F., Guo X., Jiang X. (2019). Genetic characteristics of pathogenic Leptospira in wild small animals and livestock in Jiangxi Province, China, 2002–2015. PLoS Negl. Trop. Dis..

[26] Niloofa R., Fernando N., de Silva N.L., Karunanayake L., Wickramasinghe H., Dikmadugoda N., Premawansa G., Wickramasinghe R., de Silva H.J., Premawansa S. (2015). Diagnosis of Leptospirosis: Comparison between Microscopic Agglutination Test, IgM-ELISA and IgM Rapid Immunochromatography Test. PLoS ONE.

[27] Terpstra W.J., World Health Organization, International Leptospirosis Society (2003). Human Leptospirosis: Guidance for Diagnosis, Surveillance and Control.

[28] Hartskeerl R.A., Collares-Pereira M., Ellis W.A. (2011). Emergence, control and re-emerging leptospirosis: Dynamics of infection in the changing world. Clin. Microbiol. Infect..

[29] Venkataraman K., Nedunchelliyan S. (1992). Epidemiology of an outbreak of leptospirosis in man and dog. Comp. Immunol. Microbiol. Infect. Dis..

[30] Lelu M., Muñoz-Zanzi C., Higgins B., Galloway R. (2015). Seroepidemiology of leptospirosis in dogs from rural and slum communities of Los Rios Region, Chile. BMC Vet. Res..

[31] Fahimipour A., Khaki P., Moradi Bidhendi S. (2021). Seroepidemiological Analysis of Leptospiral infection using MAT in Stray Dogs in Alborz, Iran. Arch. Razi Inst..

[32] Baranton G., Postic D. (2006). Trends in leptospirosis epidemiology in France. Sixty-six years of passive serological surveillance from 1920 to 2003. Int. J. Infect. Dis..

[33] Jansen A., Schöneberg I., Frank C., Alpers K., Schneider T., Stark K. (2005). Leptospirosis in Germany, 1962–2003. Emerg. Infect. Dis..

[34] Eric Klaasen H.L., Adler B. (2015). Recent advances in canine leptospirosis: Focus on vaccine development. Vet. Med..

