# Supplementary material for: Seroepidemiological Analysis of Canine Leptospira Species Infections in Changchun, China
Source: Pathogens. 2023 Jul 12;12(7):930. doi: 10.3390/pathogens12070930 (PMC10384461; doi:10.3390/pathogens12070930)
Supplement: Supplementary file 1 [file pathogens-12-00930-s001.zip › pathogens-2439765-supplementary.pdf]

Supplementary Table S1. Leptospira strain used in MAT.

Serogroup	Strain
Icterohaemorrhagiae	56601
Javanica	56602
Canicola	56603
Ballum	56604
Pyrogenes	56605
Autumnalis	56606
Australis	56607
Pomona	56608
Grippotyphosa	56609
Hebdomadis	56610
Bataviae	56612
Tarassovi	56613
Manhao	56615
Sejroe	56635
Mini	56655
